# Genetic Networks of Liver Metabolism Revealed by Integration of Metabolic and Transcriptional Profiling

**DOI:** 10.1371/journal.pgen.1000034

**Published:** 2008-03-14

**Authors:** Christine T. Ferrara, Ping Wang, Elias Chaibub Neto, Robert D. Stevens, James R. Bain, Brett R. Wenner, Olga R. Ilkayeva, Mark P. Keller, Daniel A. Blasiole, Christina Kendziorski, Brian S. Yandell, Christopher B. Newgard, Alan D. Attie

**Affiliations:** 1 Sarah W. Stedman Nutrition and Metabolism Center, Duke University Medical Center, Durham, North Carolina, United States of America; 2 Department of Pharmacology and Cancer Biology, Duke University Medical Center, Durham, North Carolina, United States of America; 3 Department of Biochemistry, University of Wisconsin, Madison, Wisconsin, United States of America; 4 Department of Statistics, University of Wisconsin, Madison, Wisconsin, United States of America; 5 Department of Biostatistics and Medical Informatics, University of Wisconsin, Madison, Wisconsin, United States of America; 6 Department of Horticulture, University of Wisconsin, Madison, Wisconsin, United States of America; The Wellcome Trust Sanger Institute, United Kingdom

## Abstract

Although numerous quantitative trait loci (QTL) influencing disease-related phenotypes have been detected through gene mapping and positional cloning, identification of the individual gene(s) and molecular pathways leading to those phenotypes is often elusive. One way to improve understanding of genetic architecture is to classify phenotypes in greater depth by including transcriptional and metabolic profiling. In the current study, we have generated and analyzed mRNA expression and metabolic profiles in liver samples obtained in an F2 intercross between the diabetes-resistant C57BL/6 *leptin^ob/ob^* and the diabetes-susceptible BTBR *leptin^ob/ob^* mouse strains. This cross, which segregates for genotype and physiological traits, was previously used to identify several diabetes-related QTL. Our current investigation includes microarray analysis of over 40,000 probe sets, plus quantitative mass spectrometry-based measurements of sixty-seven intermediary metabolites in three different classes (amino acids, organic acids, and acyl-carnitines). We show that liver metabolites map to distinct genetic regions, thereby indicating that tissue metabolites are heritable. We also demonstrate that genomic analysis can be integrated with liver mRNA expression and metabolite profiling data to construct causal networks for control of specific metabolic processes in liver. As a proof of principle of the practical significance of this integrative approach, we illustrate the construction of a specific causal network that links gene expression and metabolic changes in the context of glutamate metabolism, and demonstrate its validity by showing that genes in the network respond to changes in glutamine and glutamate availability. Thus, the methods described here have the potential to reveal regulatory networks that contribute to chronic, complex, and highly prevalent diseases and conditions such as obesity and diabetes.

## Author Summary

Although numerous quantitative trait loci (QTL) influencing disease-related phenotypes have been detected through gene mapping and positional cloning, identifying individual genes and their potential roles in molecular pathways leading to disease remains a challenge. In this study, we include transcriptional and metabolic profiling in genomic analyses to address this limitation. We investigated an F2 intercross between the diabetes-resistant C57BL/6 *leptin^ob/ob^* and the diabetes-susceptible BTBR *leptin^ob/ob^* mouse strains that segregates for genotype and diabetes-related physiological traits; blood glucose, plasma insulin and body weight. Our study shows that liver metabolites (comprised of amino acids, organic acids, and acyl-carnitines) map to distinct genetic regions, thereby indicating that tissue metabolites are heritable. We also demonstrate that genomic analysis can be integrated with liver mRNA expression and metabolite profiling data to construct causal, testable networks for control of specific metabolic processes in liver. We apply an *in vitro* study to confirm the validity of this integrative method, and thus provide a novel approach to reveal regulatory networks that contribute to chronic, complex, and highly prevalent diseases and conditions such as obesity and diabetes.

## Introduction

Genetic linkage and association studies have the power to establish a causal link between gene loci and physiological traits. These studies can make novel connections between biological processes that would not otherwise be predictable based on current knowledge. The pace of gene discovery has greatly accelerated in recent years, and numerous quantitative trait loci (QTL) influencing disease-related phenotypes have been identified through gene mapping and positional cloning. While it has become relatively straightforward to map a phenotype to a broad genomic region, identification of the individual gene(s) responsible for the phenotype remains difficult. Consequently, only a few percent of the many QTL that have been mapped have had their underlying gene(s) identified [1]–[7]. Another limitation of traditional QTL mapping is that it is based on association with a physiological phenotype, but often does not reveal the molecular pathways leading to that phenotype.

One way to uncover molecular mechanisms of disease states is to broadly expand the types of phenotypes analyzed in genetic screens. For example, with microarray technology, one can measure the abundance of virtually all mRNAs in a segregating sample. Importantly, mRNA abundance shows sufficient heritability in outbred populations and experimental crosses to allow mapping of gene loci that control gene expression, termed expression QTL (eQTL) [8],[9]. When eQTL co-localize with a physiological QTL, one can hypothesize a shared regulator and offer a potential pathway leading to the physiological trait [9],[10].

The pathway between a QTL and a physiological trait often involves changes in the steady-state levels of metabolic intermediates, in addition to changes in mRNA abundance. These metabolites can correlate with the genetic, transcriptional, translational, post-translational, and environmental influences on phenotype [7],[11]. Moreover, metabolites are intermediates in signaling pathways that can regulate gene expression. For example, fatty acids act as ligands for several of the PPAR nuclear hormone receptors, bile acids activate FXR in liver, and diacylglycerol regulates protein kinase C [12]–[14]. Metabolite abundance reflects a biological response to exogenous and endogenous inputs, and when investigating pathways from genotype to phenotype, metabolites can provide a powerful complement to gene expression data and give novel insights into disease pathogenesis mechanisms [7], [11], [15]–[25].

Our laboratories have begun to apply targeted metabolic profiling to study mechanisms underlying obesity-induced diabetes [15]–[20], but have not yet attempted to integrate these methods with genotyping and transcriptional profiling. This has included the application of gas chromatography/mass spectrometry (GC/MS) and tandem mass spectrometry (MS/MS) for measurements of acyl-carnitine, organic acid, amino acid, free fatty acid, and long and medium-chain acyl-CoA metabolites in tissue extracts and bodily fluids. Herein, we have applied these methods to measure various metabolites in liver samples from mouse strains that differ in susceptibility to obesity-induced diabetes.

C57BL/6 (B6) *leptin^ob/ob^* mice are obese but essentially resistant to diabetes, whereas BTBR *leptin^ob/ob^* mice are severely diabetic [22]. In an F2 cohort derived from these parental strains, we have shown that the range of blood glucose, insulin levels, and body weight exceeds that of either the C57BL/6 (B6) *leptin^ob/ob^* or BTBR *leptin^ob/ob^* parental strains. We went on to identify several diabetes-related QTL in this F2 sample [21],[22]. In the current study, we focused on a subset of 60 F2 mice that have previously been evaluated in detail with regard to liver gene expression profiles [24] to ask if the abundances of hepatic metabolic intermediates would show sufficient heritability to enable us to map metabolic QTL (mQTL). Because we previously performed mRNA expression profiling on liver samples from this F2 sample, we were also able to investigate the potential for integrative analysis of the expression profiling and metabolite data sets.

We show that liver metabolites do map to distinct genetic regions, thereby demonstrating that tissue metabolite profiles are heritable. In addition, we show that mQTL co-localize with eQTL, suggesting common genetic regulators. Finally, as a proof of principle of the practical significance of this multi-disciplinary approach, we illustrate the construction of a specific causal network that links gene expression and metabolic changes, and demonstrate its validity by targeted gene expression analysis.

## Results

### Metabolites of Similar Function Are Highly Correlated across the F2 Population

We determined the concentration of 67 liver metabolites, comprised of 15 amino acids and urea cycle intermediates, 45 acyl-carnitines, and 7 organic acids (TCA cycle intermediates and related metabolites) in the F2 sample. The specific analytes are summarized in Table S1.

**Figure 1:**
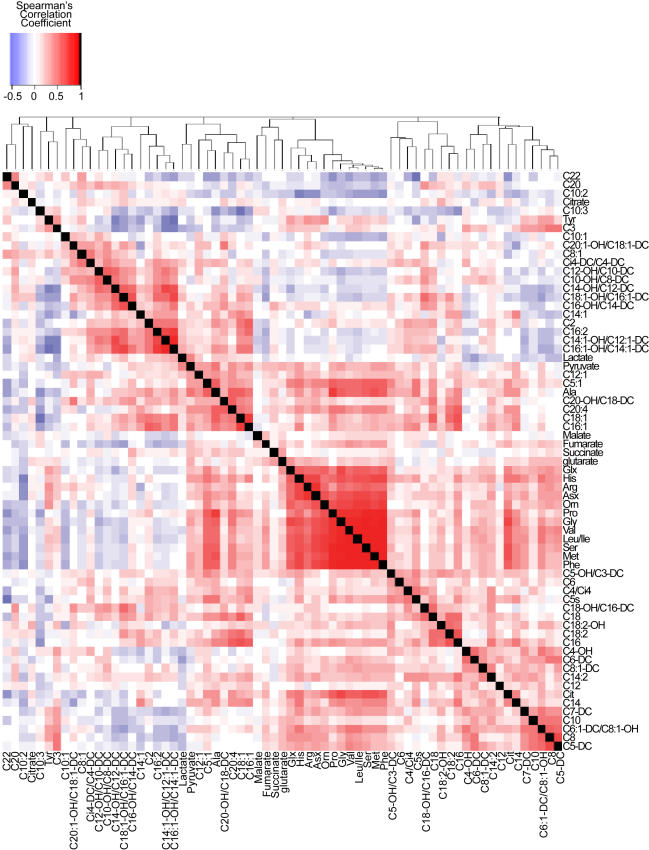
Heat map of correlations between liver metabolites. Each square represents the Spearman's correlation coefficient between the metabolite of the column with that of the row (|r|>0.254, p<0.05; |r|>0.330, p<0.01). Metabolite order is determined as in hierarchical clustering using the distance function 1-correlation. Self-self correlations are identified in black. Acyl-carnitines are annotated according to clinical acyl-carnitine profile shorthand and amino acids by three letter code; other metabolite abbreviations are found in Table S1. Individual correlation coefficients can be found in Table S2.

We created a correlation matrix of all pairwise comparisons among individual metabolites. Unsupervised hierarchical clustering revealed several “hot spots” of highly correlated metabolites (Figure 1). It is striking that several hot spots correspond to the biochemical pathway to which the metabolites belong. For example, 12 of the 15 amino acids cluster in this matrix. Moreover, when we consider pairwise correlations between all amino acids, 75% had absolute correlation coefficients greater than 0.5 (p<0.01) (Table S2). Permutation analysis of these pairwise correlations confirm that the 15 amino acids correlate as a functional group (p<0.001). Several specific acyl-carnitine derivatives are also clustered, such as hexadecadienoyl carnitine (C16∶2), 3-hydroxy-tetradecanoyl carnitine or dodecenedioyl carnitine (C14∶1-OH/C12∶1-DC), and 3-hydroxy-palmitoleoyl carnitine or *cis*-5-tetradecenedioyl carnitine (C16∶1-OH/C14∶1-DC). The fact that metabolites of a common functional group are highly correlated suggests that there are potential regulators of these biochemical pathways segregating in this F2 sample.

In another cluster, pyruvate correlates most highly with alanine (r = 0.53, p<0.01), and also with lactate and tiglyl carnitine (C5∶1) (p<0.01). Alanine and short-chain acyl-carnitines are products of peripheral protein and fatty acid catabolism, respectively, and are delivered to the liver. The liver uses alanine, along with pyruvate and lactate, as gluconeogenic substrates and rapidly interconverts these metabolites through transamination and oxidation/reduction. The clustering of these metabolites based on their relative concentration in F2 animals suggests that static metabolic profiling can be used as a marker for changes in flux through certain metabolic pathways. All metabolite-metabolite correlation coefficients are listed in Table S2.

It has been demonstrated that mRNA abundance, as determined with microarray technology, is sufficiently heritable to map QTL [7], [8], [10], [23]–[27]. Lan et. al. showed that using expression mapping, specifically in this F2 intercross, can uncover mechanisms that explain correlations between specific transcripts [8]. We therefore sought to determine if metabolite abundance, as measured in F2 liver samples by mass spectrometry, was similarly heritable. If so, resulting metabolic QTL (mQTL) could be integrated with expression QTL (eQTL) to form network models of gene expression that might ultimately help to explain diabetes susceptibility and resistance in the BTBR *leptin^ob/ob^* and B6 *leptin^ob/ob^* strains, respectively [28],[29].

**Figure 2:**
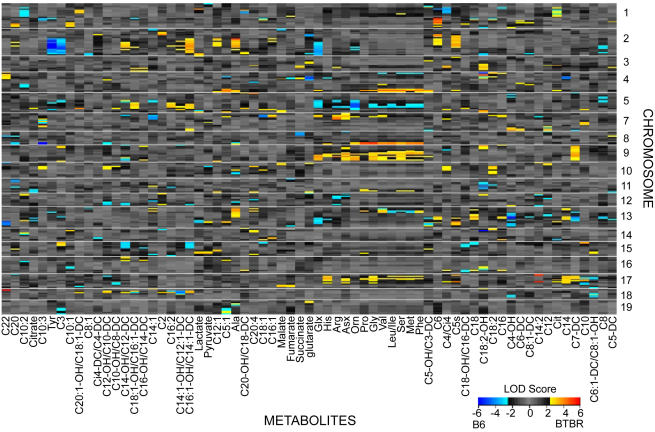
Linkage hot spots for metabolic quantitative trait loci (mQTL). Each row represents a marker; each column represents a metabolite. Metabolites are ordered as in hierarchical clustering using the distance function 1-correlation (as in Figure 1). The LOD color scale is indicated, showing blue (red) when the B6 (BTBR) allele at that marker results in an elevated level of metabolite.

We found that individual metabolites mapped to specific regions of the genome. By permutation analysis, 21% of the metabolites map significantly to genomic regions (LOD>5.0, p<0.05), indicating those genomic regions could potentially influence (either directly or indirectly) the abundance of these metabolites. We used LOD threshold of 3.0 to investigate both major and minor putative mQTL where groups of metabolites map. Figure 2 displays a heat map, with metabolites organized by hierarchical clustering as in Figure 1. The twelve amino acids that clustered based on correlation (citrulline, tyrosine, and alanine are the exceptions) map to common mQTL, e.g., an overlapping region of chromosome 9. Amino acids that act together in specific pathways show additional common mQTL. For example glx (glutamine+glutamate) and urea cycle intermediates arginine, asx (asparagine+aspartate), and ornithine, map to a common region of chromosome 7. The gluconeogenic substrates alanine and pyruvate have a mapping profile distinct from the majority of amino acids in that they lack the prominent mQTL on chromosome 9 (Figure 2). This unique alanine/pyruvate mQTL may explain why alanine clusters with pyruvate rather than the amino acids in the correlation matrix (Figure 1).

### Expected and Novel Correlations between Transcripts and Metabolites

**Figure 3:**
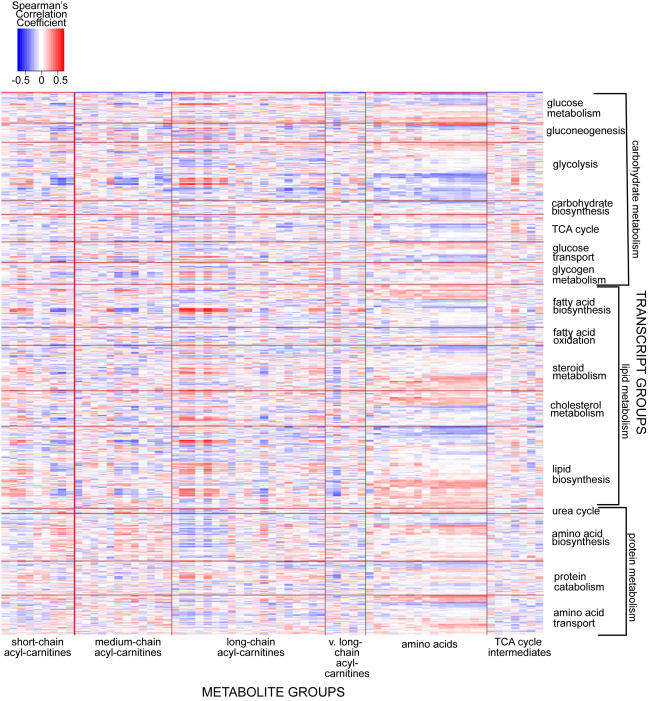
Heat map of correlations between liver metabolites and select liver transcripts. Each square represents the Spearman's correlation coefficient between the metabolite of the column with the transcript of the row (|r|>0.254, p<0.05; |r|>0.330, p<0.01). Metabolites are organized into their biochemical class; transcripts are selected based on gene ontology terms relating to biological processes in which they play a role. Correlation coefficients between individual amino acids with select transcripts are found in Table S3.

The foregoing results demonstrate that metabolites of a functional class often are correlated with one another and have common mQTL. To better understand how gene expression and metabolites are related, we adopted the approach used by Carrari [30] and created a correlation matrix between liver metabolites and selected liver transcripts of our 60 F2 mice. Three categories of transcripts were chosen, based on gene ontology terms relating to the biological process in which they play a role: 1) carbohydrate metabolism (glucose metabolism, gluconeogenesis, glycolysis, carbohydrate biosynthesis, TCA cycle, glucose transport, and glycogen metabolism); 2) lipid metabolism (fatty acid biosynthesis, fatty acid oxidation, steroid metabolism, cholesterol metabolism and biosynthesis, and lipid biosynthesis); and 3) protein metabolism (urea cycle, amino acid biosynthesis, protein catabolism, and amino acid transport). We organized the metabolites into functional classes to reveal whether biochemical groups of metabolites correlated in a specific pattern with transcripts of a particular pathway (Figure 3).

We found evidence for correlations among functionally similar metabolites and transcripts when organized by biological process. For example, several long-chain acyl-carnitine species show a positive correlation with groups of transcripts involved in glycolysis, fatty acid biosynthesis, steroid metabolism, cholesterol metabolism, and lipid biosynthesis. In contrast, a subset of medium-chain acyl-carnitines and short chain acyl-carnitines exhibit a negative correlation to these same individual transcripts. These findings are consistent with recent studies from our laboratories showing that long-chain acyl-carnitines accumulate in muscle of animals with diet-induced obesity at the expense of short-chain acyl-carnitines, and that this abnormality is resolved when obese animals are exercised [17].

The 15 amino acids displayed a common correlation pattern with mRNA transcripts in pathways of protein metabolism, as well as glycolysis, the TCA cycle, and several lipid metabolism transcripts. These amino acids are very tightly correlated with one another, leading us to investigate the role played by individual transcripts in control of amino acid abundance. Our data show that two very highly correlated metabolites often correlate with the same set of individual transcripts. However, we also see that within this metabolite group, subsets of amino acids will have a unique transcript correlation pattern (Table S3, Table S4). For example, thirteen of fifteen amino acids correlate (r>0.35, p<0.01) with *Slc38a3*, a sodium-dependent transporter that mediates entry of a select group of amino acids across the plasma membrane. There are pathways by which the few known *Slc38a3* amino acid substrates (alanine, asparagine, histidine, and glutamine) could serve as precursors for biosynthesis of non-substrate amino acids that also correlate with this transporter [31],[32]. In contrast, only valine and leucine+isoleucine correlate as highly (r>0.35, p<0.01) with *Ppargc1a* mRNA, and could represent a unique metabolic pathway involving the branched-chain amino acids.

### Correlations and Co-Mapping of Transcripts and Metabolites Produce Causal Network Models

One hypothesis that follows from our results is that unique genetic regulators could affect the abundance of clusters of metabolites. Unlike mRNA transcripts, metabolites can be interconverted with other metabolites, generating a cluster to which the precursor metabolite will be highly correlated [33]. The downstream product metabolites will also be correlated with the regulatory transcript and co-map with the eQTL of the regulatory transcript [7],[34].

Glutamate is a substrate and product in amino acid catabolic and biosynthetic pathways. Glutamate can act either as an ammonium donor or acceptor in transamination reactions (via α-ketoglutarate) and the glutamate dehydrogenase reaction, and can also be rapidly synthesized from glutamine via glutaminase, thus providing precursor metabolites for the generation of other organic acids and amino acids. Glutamine can also act as a signaling molecule to alter expression of urea cycle and gluconeogenic enzymes [35]–[39]. Given that glutamine and glutamate (glx) can generate a network of related metabolites and can also change gene expression, we focused on glx as the start-point for building a proof-of-principle causal network from the F2 liver expression and metabolite profiling data sets. We generated a network featuring glx and a limited number of transcripts that passed multiple, stringent selection filters (see materials and methods). This provided a testable network that would enable us to gain insights into metabolite-transcript relationships.

Transcript nodes of the network are highly correlated to glx (p<0.05 by 10,000 permutations) as well as other amino acids (Table 1, Table S4). Table 1 depicts the overlap of the glx mQTL interval and the physical location of the transcripts or their eQTL encompassing a 1.5 LOD support interval around LOD peaks that are at least 3.0 [40],[41]. We note that glx is correlated with mRNA of two transporters: sodium-dependent amino acid transporter *Slc38a3* and glutamate transporter *Slc1a2*, whose genes are located on chromosomes 9 (102.5 Mb) and 2 (107.5 Mb), respectively. Additionally, the glx mQTL on chromosome 9 spans a region containing *Slc38a3* and the mQTL on chromosome 2 and 9 overlaps with the eQTL of *Slc1a2* (Table 1). We hypothesize that both *Slc1a2* and *Slc38a3* could mediate the entry of glx into liver cells, but that *Slc1a2* may also have expression regulated by glx abundance.

**Table 1:** Glx network correlations and mapping.

Gene/Metabolite Symbol (Affy Primer Set)</emph>			GLX	Agxt **1418833_at**	Arg11419549_at	Asl1448350_at	Ass11416239_at	Ivd1418238_at	Pck11423439_at	Pck11439617_s_at	Slc1a21451627_a_at	Slc38a31418706_atz
***Spearman's Correlation to Glx***			*1.00*	*0.55*	*0.35*	*0.23*	*0.40*	*0.33*	*0.36*	*0.39*	*0.49*	*0.42*
**mQTL/eQTL (LOD>3±1.5 LOD) (Mb)**	**Chromosome**	1		**95.0**	13.0–171.5	13.0–156.6						
		2	114.0–178.5	68.2–178.5	145.3–171.9		**31.3**;149.7–178.4	**118.6**;68.9–151.9	**172.8**	**172.8**	**102.6**;105.2–141.8	
		3										
		4	3.5–150.1				22.6–150.1		40.8–121.5	3.6–144.6		
		5	39.3–117.7			**130.3**	47.7–129.2					
		6										
		7	13.9–35.1	13.9–28.7								
		8					48.5–119.5	46.0–170.7				36.6–100.0
		9	85.1–112.0		54.2–74.5						90.2–117.6	**107.5**
		10			**24.6**				107.2–120.4			
		11										
		12										
		13	81.4–112.0					33.1–86.4				
		14		19.2–23.1					21.1–98.3			
		15				3.3–91.8	3.3–76.0					
		16										
		17										
		18								15.5–85.7	30.7–48.3	
		19										

The Spearman's correlation coefficient of glx with the transcripts of the network is recorded (|r|>0.254, p<0.05; |r|>0.330, p<0.01). The physical location (Mb) of each transcript is noted in bold. For chromosomes containing eQTL or mQTL LOD>3.0, the transcript eQTL and glx mQTL ±1.5 LOD interval are given in Mb. Spearman's correlation coefficients between network transcripts and all fifteen amino acids are found in Table S4.

Table 1 also shows that glx is significantly correlated to argininosuccinate synthetase 1 (*Ass1*), arginase 1 (*Arg1*), phosphoenolpyruvate carboxykinase 1 (*Pck1*), isovaleryl coenzyme A dehydrogenase (*Ivd*) and alanine∶glyoxylate aminotransferase (*Agxt*) mRNAs. The physical location and/or mapping location of these transcripts with respect to the glx mQTL indicates that the metabolite-transcript relationship may go beyond correlation. For example, on chromosome 2, we see that the glx mQTL co-maps with the eQTL for *Agxt*, *Arg1*, *Ass1*, and *Ivd* [41]. This is consistent with network models in which the QTL regulates glx, which then regulates gene expression or conversely, the QTL regulates mRNA abunance of the four transcripts, which then regulate glx [9].

**Figure 4:**
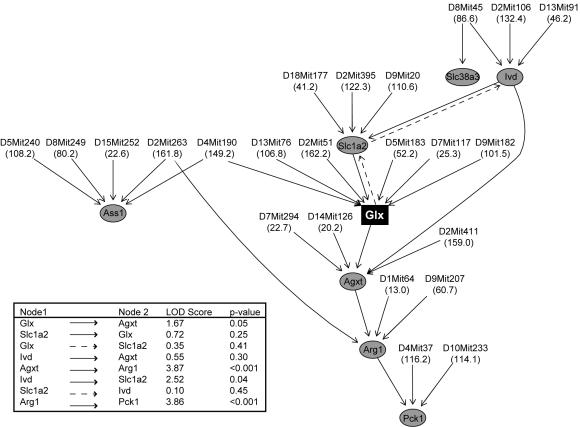
Glx network. This network consists of a select number of transcripts (grey circles) among the 250 mRNA that are most correlated to glx (black rectangle) (p<0.002). The microsatellite marker (Mb) for peak eQTL or mQTL altering levels of transcripts and metabolites, respectively, are given. For any two phenotypes connected by an edge, the direction LOD score and p-value are indicated (insert). The best solution was determined by an approximate Bayes factor (BF) and indicated in solid lines, the second best solution in dotted lines.

**Figure 5:**
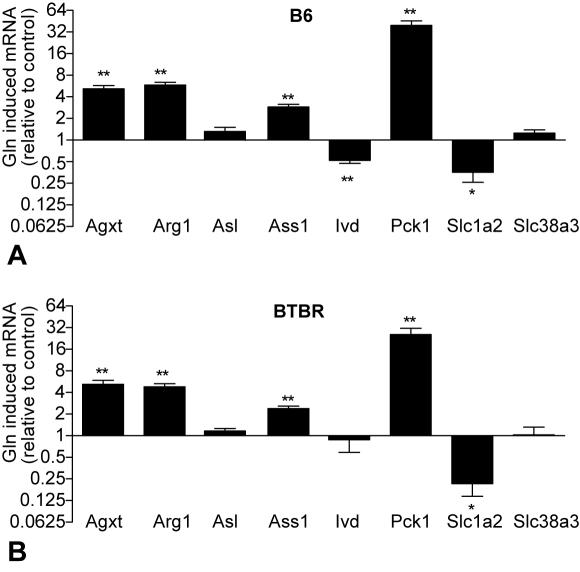
Glutamine changes hepatic gene expression. Hepatocytes from 10-week old lean B6 (A) and BTBR (B) were treated overnight +/− 10 mM glutamine (n = 5 per strain). Transcripts were measured by RT-PCR and expression was normalized to *Actb* control. Significance calculated based on the difference of delta CT value of each transcript between the untreated and glutamine treated hepatocytes for each individual animal (*p<0.05, **p<0.005).

Using the method described by Chaibub et al. (in review), we generated a causal network consisting of glx and these highly correlated transcripts (FDR = 0.014), incorporating mQTL and eQTL to determine directionality between the nodes (Figure 4). This network model predicts that modulation of glutamine and/or glutamate levels should lead to a change in the expression of *Agxt*, *Arg1*, and *Pck1*. To test this prediction, we isolated hepatocytes from lean B6 and BTBR parental strains and measured changes in gene expression as a result of addition of 10 mM glutamine to the cultured cells. Glutamine exposure changed transcript abundance, and no transcript-specific strain differences in glutamine effect on gene expression were found (p = 0.53) (Figure 5). Glutamine significantly increased expression of *Agxt*, *Arg1*, *Pck1*, and *Ass1* in both strains (p<0.05 for both strains); the increases in *Pck1* and *Ass1* confirm prior studies [36]–[39].

Given its role as a glutamate transporter, it is not surprising that *Slc1a2* is upstream of glx in the best proposed causal network (BF = 163) (Figure 4, solid lines). However, glutamine exposure in vitro reduced *Slc1a2* expression in isolated hepatocytes from either mouse strain, supporting the second-best causal network solution (Figure 4, dotted lines). Glutamine also reduced *Ivd* expression in the B6 strain but showed no effect in the BTBR strain, despite *Ivd* being upstream of glx in our best causal network. Our causal network predicts *Slc38a3* should be unchanged by glutamine treatment. Our hepatocyte experiments confirm this prediction (Figure 5). Argininosuccinate lyase (*Asl*), which is neither correlated nor co-maps with glx, served as a negative control and indeed was not altered by glutamine treatment.

## Discussion

Genomics, transcriptomics, proteomics, and metabolomics have delivered large arrays of data, allowing one to correlate physiological states with patterns of gene expression, protein levels, and metabolite abundance. A major challenge in the analysis and interpretation of this data is delivering models of causation from correlations [9],[42]. Mouse models of diabetes provide a unique method for exploring correlation structure since metabolic dysregulation creates a window for simultaneous application of multiple “omic” technologies.

We have previously shown that diabetes traits show strong heritability in an F2 intercross between the diabetes-resistant C57BL/6 *leptin^ob/ob^* and the diabetes-susceptible BTBR *leptin^ob/ob^* mouse strains. We assume that the disease phenotype is brought about by a complex pattern of gene expression changes in key tissues [21],[22]. However, we also recognize the complexity inherent in discriminating the gene expression changes that cause diabetes from those that occur as a consequence of the disease. For example, many genes are known to be responsive to elevated blood glucose levels [43]. Through correlation alone, it is difficult to distinguish these “reactive” genes from ones that are “causal” for the disease.

We have taken advantage of the high heritability of mRNA abundance phenotypes, and via microarray technology, have mapped gene loci controlling gene expression at the genome-wide level [8]. This establishes at least one node in a network simply because genetic variation leads to changes in gene expression and not *vice versa*. However, it does not establish whether the link between a locus and a phenotype is direct or via multiple steps and pathways [27],[44].

The purpose of the current study was to explore the possibility that the levels of metabolites in tissues are sufficiently heritable in an F2 intercross to provide significant linkage signals, leading to metabolic QTL. Given that many pathways converge upon common metabolites and that these pathways have multiple controllers, any one genetic locus may not alter metabolite levels significantly, and therefore may not be identified as a metabolite QTL. Nonetheless, in our F2 sample, we found significant linkage signals, including some that are quite strong (*e.g.* tyrosine: LOD>7, p<0.005; chromosome 2).

Our results reveal that metabolites can be mapped to distinct genetic regions, much like mRNA transcripts. Although QTL mapping in an F2 sample does not provide sufficient resolution to identify individual genes with high certainty, it can yield novel information about regulatory networks. Phenotypes mapping to the same locus can be hypothesized to be co-regulated by that locus. With our definition of “phenotype” now including transcripts, metabolites, and physiological traits, we can begin to devise relationships between these phenotypes and genetic regions.

This F2 study provides evidence of co-regulation of biologically related pathways. An example is the correlations we found between amino acids and short-chain acyl-carnitine derivatives. These findings are consistent with our understanding of metabolic physiology. In a catabolic, “glucose starved” state, muscle degrades proteins and delivers amino acids to the liver for glucose production. The liver transaminates amino acids to corresponding α-keto acid gluconeogenic substrates. Alpha-ketoglutarate is often the α-keto acid acceptor for these transaminase reactions, generating glutamate as a product. Glutamate, which can also be generated from glutamine in the glutaminase reaction, is then deaminated to produce ammonia by glutamate dehydrogenase, to be fixed through the urea cycle. Additionally, hepatic fatty acid oxidation and amino acid catabolism yield even and odd-numbered short-chain acyl CoAs, which can be used for fuel and for production of ketone bodies. These short-chain acyl-CoA species are readily converted to the cognate carnitine esters, which we have profiled by MS/MS in this study.

The amino acid metabolites provide the most striking evidence of functional clustering. We see in both the correlation matrix (Figure 1) and the genetic linkage data (Figure 2) that the majority of amino acids group together. However, a subset of the amino acids, asx, glx, arginine, and ornithine uniquely map to chromosome 7. Our data predict that these metabolites are driven by different genetic regulators, leading to a unique mapping signature, even within a group of highly correlated metabolites. The C/EBP transcription factors have been shown to alter expression of enzymes acting in the urea cycle and gluconeogenic pathway [45]–[51], and the C/EBPα isoform is encoded on chromosome 7. Although we cannot determine that metabolites are mapping to the same individual genes, we can identify genetic regions that coordinate groups of metabolites and transcripts and contain plausible candidate genes.

The relationship between mRNA transcripts and metabolites, however, can be bi-directional. Our network identifies a specific metabolite, glx that regulates gene expression. This is consistent with previous studies where glutamine alone increases hepatic expression of argininosuccinate synthetase and phosphoenolpyruvate carboxykinase, but when combined with other essential amino acids, alters additional transcripts of urea cycle and gluconeogenic pathways [36]–[38],[52]. Our work extends these prior observations by showing that glutamine also changes expression of *Agxt*, *Arg1*, *Ivd*, and *Slc1a2*, but does not alter *Slc38a3*, despite the positive correlation with this transcript. The combination of pathway construction based on transcriptional and metabolic profiling and direct model testing in living cells provides evidence for a new pathway by which glx can regulate a key gluconeogenic enzyme. Future studies will be needed to investigate if this pathway is perturbed in development of diabetes.

The glutamine induced reduction in *Slc1a2* expression was unexpected given that this glutamate transporter is upstream of glx in the best-proposed causal network (Figure 4, solid lines). *Slc1a2* mRNA abundance, however, maps in *trans* (to a locus distinct from the physical location of the gene) to chromosome 9, its eQTL overlapping with the glx mQTL. It is therefore possible that glutamine could regulate *Slc1a2*, as indicated by the second causal network (Figure 4, dotted lines). Several studies have shown that *Slc1a2* expression in astrocytes is reduced by increased ammonia [45]–[47], [51], [53]–[55]. Despite the positive correlation between *Slc1a2* and glx in vivo, the glutamine-treated hepatocytes produce ammonia via glutaminase, and could decrease expression of hepatic *Slc1a2* in vitro. We also did not predict altered expression of *Ivd*, an enzyme of leucine oxidation. It is interesting to note that *Ivd* is a case where a gene maps both in *cis* (to the locus containing the *Ivd* gene) and in *trans*, here overlapping with the glx mQTL on chromosomes 2 and 13. Studies have shown that glutamine has an inverse relationship with leucine oxidation, and this could be mediated by glutamine-induced decreased *Ivd* expression [48],[50].

We show that the combined use of eQTL and mQTL, with correlations allows one to derive a network and establish data-driven hypotheses about metabolite and gene expression relationships. For example, glycine and serine are the two amino acids most highly correlated with glx, and the transcript most highly correlated with glx is *Agxt* (Table 1, Table S2). Indeed, in our experiments, *Agxt* was upregulated by glutamine. We hypothesize that the upregulation by glx of *Agxt* is one mechanism by which glx is correlated with glycine and serine since *Agxt* catalyzes the transamination of glyoxalate to form glycine, which can then be converted to serine. In further support of this hypothesis, in the F2 sample, serine and glycine correlate (r>0.5, p<0.01) to *Agxt*.

The concurrent use of transcriptomics and metabolomics is not limited to one biochemical pathway. For example, the correlation between amino acids and transcripts of carbohydrate and lipid metabolism might reflect a broader signaling function of amino acids beyond pathways of protein metabolism. Furthermore, this correlation, co-mapping, and causal network analysis can uncover roles for transcripts of unknown function. We note Riken clones and ESTs are among the transcripts highly correlated to individual metabolites (Table S3). By incorporating these transcripts of unknown function as nodes into causal networks, along with transcripts from known pathways, we may infer the functions of these previously unidentified mRNA species.

In conclusion, this study shows that metabolites, in addition to transcripts and physiological traits, can be mapped to genetic regions, providing a powerful tool to establish connections between genetic loci and physiological traits. The groups of metabolites and transcripts that are correlated or co-map to physiological traits in our F2 sample may offer insight into metabolic pathways that are causal or reactive to diabetes pathology.

## Materials and Methods

### Animals

BTBR, B6, and B6-*ob/+* mice were purchased from The Jackson Laboratory (Bar Harbor, ME) and bred at the University of Wisconsin. The lineage and characteristics of the BTBR strain have been reviewed by Ranheim et al. Mice were housed in an environmentally controlled facility (12-hour light and dark cycles) and were weaned at 3 weeks of age onto a 6% fat diet (Purina; #5008). Mice had ad libitum access to food and water, except for 4 hour fasting periods before blood draws and killing (by CO_2_ asphyxiation). Plasma glucose levels were measured using a commercially available kit (994-90902; Wako Chemicals). Plasma insulin levels were measured by radioimmunoassay (RI-13K; Linco Research).

The facilities and research protocols were approved by the University of Wisconsin Institutional Animal Care and Use Committee.

### Genotyping

Sixty F2 *leptin^ob/ob^* mice ranging in age from 13 to 26 weeks were genotyped as previously described [22]. Mapmaker/EXP was used to compile genotype data into framework map.

### RNA Collection and Microarray

Liver RNA was arrayed as described in Lan et. al [8]. Ten to 12 week old male and female F2 *leptin^ob/ob^* mice were killed by CO_2_ asphyxiation after a 4-h fast. Total RNA from sixty F2 mice using RNAzol reagent (Tel-Test) and was further purified using an RNeasy kit (Qiagen). The sample labeling, microarray hybridization, washing, and scanning were performed according to the manufacturer's protocols (Affymetrix). Labeled cRNA was prepared and hybridization assay procedures including preparation of solutions were carried out as described in the Affymetrix GeneChip Expression Analysis Technical Manual. A total of 60 MOE430A and MOE430B arrays were used to monitor the expression levels of approximately 45,000 genes or ESTs. The distribution of fluorescent material on the array was obtained using G2500A GeneArray Scanner (Affymetrix). Microarray Suite (MAS) version 5.0 and GeneChip Operating Software (GCOS) supplied by Affymetrix was used to perform gene expression analysis. Expression levels of all the transcripts were estimated using the RMA algorithm [49].

### Liver Metabolite Quantification

Amino acids, acyl-carnitines and organic acids were measured using stable isotope dilution techniques [15],[18],[56]. Amino acids and acyl-carnitine species were measured using flow injection tandem mass spectrometry and sample preparation methods described previously [15],[56]. Briefly, samples were equilibrated with a cocktail of internal standards, de-proteinated by precipitation with methanol, aliquoted supernatants were dried, and then esterified with hot, acidic methanol (acyl-carnitines) or *n*-butanol (amino acids). The data were acquired using a Micromass Quattro micro TM system equipped with a model 2777 autosampler, a model 1525 µ HPLC solvent delivery system and a data system controlled by MassLynx 4.0 operating system (Waters, Milford, MA) [15],[56]. Organic acids were quantified using a previously described method that utilizes Trace GC Ultra coupled to a Trace DSQ MS operating under Excalibur 1.4 (Thermo Fisher Scientific, Austin, TX) [18].

Sixty-seven liver metabolites were measured, comprised of 15 amino acids and urea cycle intermediates, 45 acyl-carnitine derivatives, and 7 organic acids (TCA cycle intermediates and related analytes). The specific metabolites are listed in Table S1. All MS analyses employed stable-isotope-dilution. The standards serve both to help identify each of the analyte peaks and provide the reference for quantifying their levels. Quantification was facilitated by addition of mixtures of known quantities of stable-isotope internal standards from Isotec (St. Louis, MO), Cambridge Isotope Laboratories (Andover, MA), and CDN Isotopes (Pointe-Claire, Quebec, CN) to samples, as follows: Acyl-carnitine assays–D_3_-acetyl, D_3_-propionyl, D_3_-butyryl, D_9_-isovaleryl, D_3_-octanoyl, and D_3_-palmitoyl carnitines; Amino acid assays–^15^N_1_,^13^C_1_-glycine, D_4_-alanine, D_8_-valine, D_7_-proline, D_3_-serine, D_3_-leucine, D_3_-methionine, D_5_-phenylalanine, D_4_-tyrosine, D_3_-aspartate, D_3_-glutamate, D_2_-ornithine, D_2_-citrulline, and D_5_-arginine; Organic acid assays–D_3_-lactate, D_3_-pyruvate, ^13^C_4_-succinate, D_2_-fumarate, D_4_-glutarate, ^13^C_1_-malate, D_6_-*alpha*-ketoglutarate, and D_3_-citrate. In addition to mass, analytes are identified on the basis of the particular MS/MS transitions that we monitor for each class of metabolites. For example, all acyl-carnitine methyl esters produce a fragment m/z 99. We make the assumption that all even mass precursors ions of m/z 99 are acyl-carnitines to which we assign plausible molecular structures. We differentiate isobaric structures e.g., dicarboxylic and hydroxylated acyl-carnitines, by comparing of MS/MS spectra for precursors of m/z 85 butylated acyl-carnitine species. We can infer whether the original compound had one or two carboxyl groups on the basis of the mass change from methyl to butyl esters.

Given our sample size, we initially analyzed metabolite abundance by hierarchical clustering using the distance function 1-correlation [40], [57]–[60]. Pairwise Spearman correlation coefficients of r>0.254 and r>0.330 reflected p-values p<0.05 and p<0.01, respectively. To test whether the 15 amino acids are significantly correlated as a group, groups of 15 metabolites were permuted 1,000 times and the percentage of pairwise correlations exceeding 0.5 was recorded for each group. The fifteen amino acids cluster significantly as a group based on 1,000 permutations (p<0.001).

### QTL Analysis

Detection and mapping of QTL was performed as previously described [8],[22]. Briefly, genotypes of 512 F_2_ mice at 293 markers were assembled using MAPMAKER/EXP [61]. A previously established subset of 60 mice with transcript data was used for expression QTL analysis [24]. Interval mapping methods adjusted for sex as implemented in R/qtl [62] were used to compute linkage to the traits of interest and to investigate mode of inheritance. The traits included the 45,265 probe sets surveyed by microarray analysis, and the 67 liver metabolites assayed by MS methods.

We used standard interval mapping implemented in R/qtl to map each of the transcripts and liver metabolites at 1-cM resolution with age as additive covariates and sex as both additive and interactive covariates [62]. A LOD threshold of 5.0 is required to reach a level of p<0.05 in this data set with sample size 60 based on 10,000 permutations. We used threshold of 3.0 in order to highlight genetic regions to which groups of metabolites map. To visualize regions of mQTL co-localization in highly correlated metabolites (Figure 2), we constructed heat maps where metabolites are ordered as in hierarchical clustering using 1-correlation, as in Figure 1. When mice with the B6 allele at a marker have greater levels of metabolites on average than mice with the BTBR allele at that marker, the LOD score at that marker is multiplied by −1. This adjustment allows us to visualize whether the B6 or BTBR allele results in elevated metabolite abundance.

### In Vitro Hepatocyte Experiments

Hepatocytes from 10-week lean male and female BTBR and B6 parental strain mice (n = 5 for each genotype) were isolated by liver perfusion [63]. Hepatocytes were seeded at subconfluency (3.5 × 10^6^ cells/6 well plate) in low glucose DMEM (GIBCO) supplemented with FBS (10% vol/vol; GIBCO), pen/strep antibiotic (1%, GIBCO), glutamine (2 mM; GIBCO), and pyruvate (1 mM; GIBCO). Cells were left to attach for 3 hours in an incubator at 37°C, 5% CO_2_. After a wash with PBS, the cells were treated with unsupplemented DMEM (Sigma) with 1 g/L glucose, pen/strep (1%), and +/− 10 mM glutamine. Cells were treated for 24 hours.

RNA was extracted from hepatocytes using RNeasy kits (Qiagen) after treatment described above. Hepatocytes in 6-well plates were homogenized in 0.35 ml of RLT buffer and stored at −80 C. RNA was purified using RNeasy-mini columns (Qiagen) according to the manufacturer's directions. The ratio of the optical densities from RNA samples measured at 260 and 280 nm was used to evaluate nucleic acid purity and total RNA concentrations were determined by the absorbance at 260 nm. The quality of total RNA was estimated based on the integrity of 28S and 18S rRNA separated using 1% agarose gel electrophoresis.

Gene expression was measured using a 7500 fast real-time PCR system (Applied Biosystems). cDNA was synthesized from 1 ug of total RNA using the SuperScriptIII first-strand cDNA synthesis kit (Invitrogen) primed with a mixture of oligo-dT and random hexamers. Primers were obtained from Integrated DNA Technologies and MWG Biotechnology. The SYBR Green PCR core reagent kit (Applied Biosystems) was used to determine relative expression. The housekeeping gene *Actb* was used as a normalization control. Primer sequences and gene accession codes for transcripts of the glx network are provided in Table S5.

### Networks

Causal networks were constructed using the methods of Chaibub, et al. (in review). Although the network has the ability to accommodate 100 or more transcripts, we chose a limited number of transcripts passing several selection filters. The transcripts for the glx network were derived from the top 250 most correlated transcripts (p<0.002) according to the WebQTL software (www.genenetwork.org). A hypergeometric test was performed and identified the GO term category “metabolism” as one of the two processes significantly enriched by these correlates (p<0.004). Transcripts were chosen from this category, with an additional requirement being that they have at least one eQTL overlapping with the glx mQTL (Table 1). QTL in the genetic region encompassing a 1.5 LOD support interval around LOD peaks that are at least 3.0 are also included [41],[42]. Based on 10,000 permutations for each of the transcripts, the LOD threshold is significantly higher to reach significance (LOD>5.0 is required for p<0.05), but the 3.0 threshold was used include major and minor putative QTL [8],[24].

If more than one probe set was used to identify a transcript of interest, only probe sets with a grade A annotation on Affymetrix were considered. For these probe sets, only those with all eleven oligonucleotides aligning (via BLAST) to their appropriate target sequence provided by the National Center for Biotechnology Information (www.ncbi.nlm.nih.gov) were considered acceptable. If more than one primer set still identified the transcript, an average of the probe sets in the network.

We built an undirected dependency graph (UDG) of order 6 with glx and these transcripts as nodes with a two-tailed significance level of 0.05 [64]. We remove edges that are based on spurious or partial correlations, and then orient causal edges between all pairs of connected phenotypes using associated multiple QTLs to break likelihood equivalence. Quantitative trait loci for glx and the selected transcripts were identified with R/qtl [62] using a 3.0 LOD cutoff; the marker closest to each peak provided key information for inferring causal direction. We oriented phenotype edges using our QTL-directed dependency graph (QDG) approach. For any two phenotypes connected by an edge, the direction LOD score was computed by regressing these phenotypes on each other and on their respective multiple QTLs, adjusting for age and for QTL-sex interactions, and by other phenotypes that might be directly connected to either phenotype by an UDG edge. For each edge, we evaluate a LOD score comparing the two possible orientations and we orient the edge in favor of the direction with the higher likelihood in the ratio. P-values for the direction of the edges were computed using 10,000 permutations. Our QDG algorithm used random starts to converge to possible solutions. The best solution was determined by an approximate Bayes factor (BF) [65],[66]. A detailed materials and methods section describing the construction of causal networks is provided in Supporting Protocols (Protocol S1).

We estimated network parameters from the true data and simulated synthetic data according to the causal network in Figure 4. We simulated 1,000 realizations from the causal network and for each edge, we recorded the percentage of undirected edges recovered by the UDG algorithm and the percentage correctly inferred direction by the orientation steps of the QDG algorithm. Overall, the average percentage of true recovered edges was 75% and the average percentage of correctly inferred direction was 83%. False edges were detected at a rate below 2%. To calculate the false discovery rate for the network, we simulated 1,000 data sets from the true network. For each data set, the UDG algorithm was used to infer the network topology, and computed the fraction of false edges (those detected that do not exist in the true network) relative to the total number edges detected by the UDG algorithm. The FDR for the network topology, computed as the average fraction for these 1,000 simulations, is 0.014.

### Gene Expression Analysis

The fold changes relative to the untreated hepatocytes for each animal were calculated. An overall ANOVA analysis was performed with gene transcripts nested within subject; interest focused on gene transcript effects and possible gene transcript differences between strains. This analysis showed that glutamine-induced expression change differed by gene (p<0.0001). Significant overall gene transcript effects allows separate transcript-specific paired t-tests between the difference in delta CT values of untreated and glutamine induced gene expression (relative to *Actb*) in each strain separately. Statistics on these data were analyzed with Prism software version 4.02 (Graph Pad Software) and the aov command in R (www.r-project.org).

## Supporting Information

Protocol S1 Causal network reconstruction.
(0.05 MB DOC)

Table S1 Metabolite codes. Acyl-carnitines are annotated according to clinical acyl-carnitine profile shorthand; conventional amino acids are annotated by their three-letter code. In the cases where two acyl-carnitine derivatives were unable to be distinguished by MS analysis alone, both are reported, indicating that either analyte could be the predominant species.
(0.02 MB XLS)

Table S2 Metabolite correlation matrix. All pairwise correlations between the 67 metabolites were calculated and recorded as the Spearman's correlation coefficient (|r|>0.254, p<0.05; |r|>0.330, p<0.01). Metabolites are ordered according to the strength of their correlation as in Figure 1.
(0.10 MB XLS)

Table S3 Correlation of amino acids and individual transcripts metabolic pathways. Transcripts that had Spearman's correlation r>0.4 to any individual amino acid are included (|r|>0.254, p<0.05; |r|>0.330, p<0.01). The physical location of the gene is provided (Mb). Amino acids are ordered as in Figure 3.
(0.81 MB XLS)

Table S4 Glx network correlations. The Spearman's correlation coefficient of the 15 individual amino acids with the transcripts of the glx network is recorded (|r|>0.254, p<0.05; |r|>0.330, p<0.01). Amino acids are ordered as in Figure 3.
(0.02 MB XLS)

Table S5 Primer sequences. Integrated DNA Technologies (Agxt, Pck1, Slc38a3) and MWG Biotechnology (Actb, Arg1, Asl, Ass1, Ivd, Slc1a2).
(0.02 MB XLS)
