# Correction: Genetic Networks of Liver Metabolism Revealed by Integration of Metabolic and Transcriptional Profiling

**DOI:** 10.1371/annotation/7989839d-0677-4f59-a218-f4ebb6fd0b66

**Published:** 2008-07-17

**Authors:** Christine T. Ferrara, Ping Wang, Elias Chaibub Neto, Robert D. Stevens, James R. Bain, Brett R. Wenner, Olga R. Ilkayeva, Mark P. Keller, Daniel A. Blasiole, Christina Kendziorski, Brian S. Yandell, Christopher B. Newgard, Alan D. Attie

The affiliation for the 13th author Alan D. Attie was incorrect. The correct affiliation is: Department of Biochemistry, University of Wisconsin, Madison, Wisconsin, United States of America

