# Supplementary material for: Genetic Networks of Liver Metabolism Revealed by Integration of Metabolic and Transcriptional Profiling
Source: PLoS Genet. 2008 Mar 14;4(3):e1000034. doi: 10.1371/journal.pgen.1000034 (PMC2265422; doi:10.1371/journal.pgen.1000034)
Supplement: Protocol S1 — Causal network reconstruction. (0.05 MB DOC) [file pgen.1000034.s001.doc]

**Protocol S1. Causal Network Reconstruction**

Chaibub et al. (2007) introduced a novel causal network reconstruction method for genetical genomics experiments termed QTL-directed dependency graph (QDG). Many current methods require network nodes consisting of genes physically located within the QTL support interval of other transcripts [1-5] or require common QTLs [6]. We drop these requirements, permitting construction of causal networks among phenotypes that may not have common QTLs (allowing the use of phenotypes other than age and gene expression. The QDG method is composed of two parts: (1) build an undirected graph that infers associations among phenotypes using an undirected dependency graph (UDG) [7,8]; (2) use a LOD score to determine causal direction for every edge that connects a pair of phenotypes, conditional on connected QTLs. Next we present the rational behind the proposed causal orientation method and a description of the QDG algorithm.

The goal is to causally orient every edge connecting a pair of phenotypes in an undirected network. That is, does phenotype *y_1_* drive phenotype *y_2_*, or *vise versa*?

*M_1_: y_1_* 🡪 *y_2_*  *M_2_: y_1_* 🡨 *y_2_*

Model selection procedures cannot distinguish between *M_1_* and *M_2_* because they are distribution or likelihood equivalent, eg. *f*(*y_1_*)*f*(*y_2_|y_1_*)=*f*(*y_1_,y_2_*)=*f*(*y_2_*)*f*(*y_1_|y_2_*). Including QTL with genotypes **q_1_** affecting *y_1_* and **q_2_** affecting *y_2_*, we can resolve direction because new models

*M*_1_:* **q_1_** 🡪 *y_1_* 🡪 *y_2_* 🡨**q_2_** *M*_2_:* **q_1_** 🡪 *y_1_* 🡨 *y_2_* 🡨**q_2_**

are *not* likelihood equivalent since the predictive densities disagree, *f*(**q_1_**)*f*(*y_1_*|**q_1_**)*f*(*y_2_|y_1_,***q_2_**)*f*(**q_2_**)≠ *f*(**q_2_**)*f*(*y_2_|***q_2_**)*f*(*y_1_|y_2_****,*q_1_**)*f*(**q_1_**). Therefore, we distinguish between models *M*_1_* and *M*_2_* using a LOD score that conditions on genotypes at multiple QTL (derived from earlier gene mapping of phenotypes). Edge orientation among pairs of phenotypes is based on a linear regression model with phenotypes regressed on QTL genotypes and on additive or interacting covariates such as sex, age, and other phenotypes. We orient each edge, condition on all other nodes (phenotypic, genotypic, or covariate) that are connected to that edge. For each edge, we evaluate a LDO score comparing the two possible orientations. We orient the edge in favor of the direction with the higher likelihood in the ratio. For the simple network presented above, this ratio is a contrast of LODs,

*LOD=*(*LOD_1_+LOD_2|1_*)- (*LOD_2_+LOD_1|2_*)

With LOD scores comparing the “full” model (a multiple QTL model possibly containing covariates and interactions) with the “reduced” model (no QTL or covariates):

*LOD_1_ = log_10_* {П_i_ *f*(*y_1i_|***q**_1i_) */ f*(*y_1i_*)}*, LOD_2|1_ = log_10_* {П_i_ *f*(*y_2i_|y_1i_,***q**_2i_) */ f*(*y_2i_*)}*,*

*LOD_2_ = log_10_* {П*_i_ f*(*y_2i_|****q****_2i_*) */ f*(*y_2i_*)}*, LOD_1|2_ = log_10_* {П_i_ *f*(*y_1i_|y_2i_* **q**_1i_) */ f*(*y_1i_*)}*.*

In more complex networks, we orient edges in two steps: (1) build an initial directed network orienting each edge as above; (2) recomputed the LOD score for each edge connecting a pair of phenotype nodes by condition on all other phenotypes causative to either or both nodes. We repeat the second step for all edges until no edge switches direction and this involves some iteration to find the best orientation across the entire graph. This algorithm may find more than one solution. That is, starting with the algorithm from a different edge ordering may yield a different graph. In order to get the best solution we: (1) re-run this algorithm 1,000 times using different initial edges to get alternative solutions; (2) score each solution using the maximized log-likelihood; (3) select the graph with the highest score. The strength of evidence for each edge direction is evaluated by p-values based on 10,000 permutations.

We provide supporting simulations in Chaibub et al. (in review) that demonstrate this method recovers and correctly orient the edges with high probability.

**References**

1. Bing N, Hoeschele I (2005) Genetical genomics analysis of a yeast segregant population for transcription network inference. Genetics 170: 533-542.

2. Jansen RC, Nap JP (2001) Genetical genomics: the added value from segregation. Trends Genet 17: 388-391.

3. Keurentjes JJ, Fu J, Terpstra IR, Garcia JM, van den Ackerveken G, et al. (2007) Regulatory network construction in Arabidopsis by using genome-wide gene expression quantitative trait loci. Proc Natl Acad Sci U S A 104: 1708-1713.

4. Kulp DC, Jagalur M (2006) Causal inference of regulator-target pairs by gene mapping of expression phenotypes. BMC Genomics 7: 125.

5. Zhu J, Lum PY, Lamb J, GuhaThakurta D, Edwards SW, et al. (2004) An integrative genomics approach to the reconstruction of gene networks in segregating populations. Cytogenet Genome Res 105: 363-374.

6. Schadt EE, Lamb J, Yang X, Zhu J, Edwards S, et al. (2005) An integrative genomics approach to infer causal associations between gene expression and disease. Nat Genet 37: 710-717.

7. de la Fuente A, Bing N, Hoeschele I, Mendes P (2004) Discovery of meaningful associations in genomic data using partial correlation coefficients. Bioinformatics 20: 3565-3574.

8. Shipley B (2000) Cause and Correlation in Biology. New York: Cambridge University Press.
